# Workforce Sustainability in Audiology: Evidence, Challenges, and a Roadmap to Autonomy

**DOI:** 10.1055/s-0046-1825786

**Published:** 2026-10-06

**Authors:** Victor Bray, Amyn M. Amlani

**Affiliations:** 1 Osborne Audiology Department, College of Nursing and Health Professions, Drexel University, Elkins Park, Pennsylvania, United States; 2 Department of Communication Sciences and Disorders, College of Allied Health, University of Oklahoma Health Campus, Oklahoma City, Oklahoma, United States

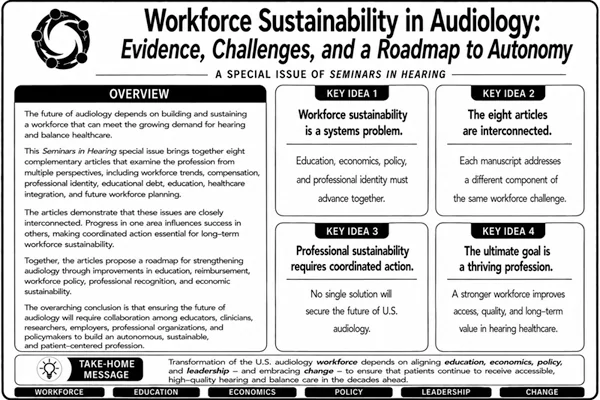

*“The professional doctorate represents the logical progress of audiology into a free-standing, autonomous profession. The AuD…assures the delivery of the highest standard of care and service to the hearing-impaired consumer.” 1*

Audiology is a doctoral-level healthcare profession responsible for the diagnosis, evaluation, treatment, and management of hearing and balance disorders. These conditions rarely occur in isolation. Untreated, hearing and balance disorders are strongly associated with a range of comorbidities—including cognitive decline and dementia, 2 increased fall risk, 3 cardiovascular disease, 4 diabetes, 5 depression, 6 and social isolation 7 —that amplify individual morbidity and contribute to downstream healthcare utilization across the lifespan. As a result, unmet hearing and balance needs represent not only a sensory health gap, but an expanding public health challenge—one that places increasing demands on the capacity and sustainability of the audiology workforce.

Because audiologic services are essential across the lifespan, the profession carries an inherent responsibility to maintain a workforce capable of meeting population needs. As Bray and Amlani have noted, “The responsibility of the profession…is to make certain it generates a dynamic, balanced, and viable supply of professionals (i.e., workforce) that can service the hearing healthcare needs of its population.” 8

This responsibility took on renewed urgency in the late 20th and early 21st centuries, when respected leaders championed the transition from a master's degree to the Doctor of Audiology (AuD). That movement was driven by a clear purpose: to elevate clinical preparation, align audiology with other doctoring health professions, and secure the professional autonomy necessary to deliver comprehensive hearing and balance care. Although the AuD has served as the entry-level credential for nearly two decades, important questions remain regarding whether the educational model—and the surrounding professional infrastructure—has fully realized the autonomy envisioned by the early architects of the profession's transformation.

Despite the successful transition to doctoral-level entry, audiology has not yet achieved the workforce scale, economic alignment, or regulatory authority typically associated with autonomous healthcare professions. As the profession approaches two decades under the AuD framework, the time is appropriate to examine several fundamental questions:

- Is audiology producing a workforce sufficient to meet current and future demand for hearing and balance care?
- How does audiology's workforce compare with other health professions in size, growth, earnings/wages, and retention?
- Has the current AuD education and training model achieved its original aims of professional autonomy, healthcare parity, and high-quality care?
- What structural changes are required to ensure long-term sustainability within an evolving healthcare landscape?

This issue of *Seminars in Hearing* does not prescribe a singular reform. Instead, it examines how multiple interdependent systems—including education, workforce dynamics, economic viability, regulatory structures, and federal classification—collectively shape whether audiology can function as a sustainable, autonomous healthcare profession. By evaluating these systems in concert, this issue seeks to clarify audiology's current workforce trajectory, identify areas of structural vulnerability, and outline a coherent pathway toward a stable, autonomous, and future-ready profession.

## Workforce Model

To investigate these questions, we developed the workforce model presented in Fig. 1 , which serves as the analytic foundation for this special issue. The model is intentionally structured to evaluate not only workforce sufficiency, but also the conditions required for audiology to function as a clinically autonomous healthcare profession.

**Fig. 1:**
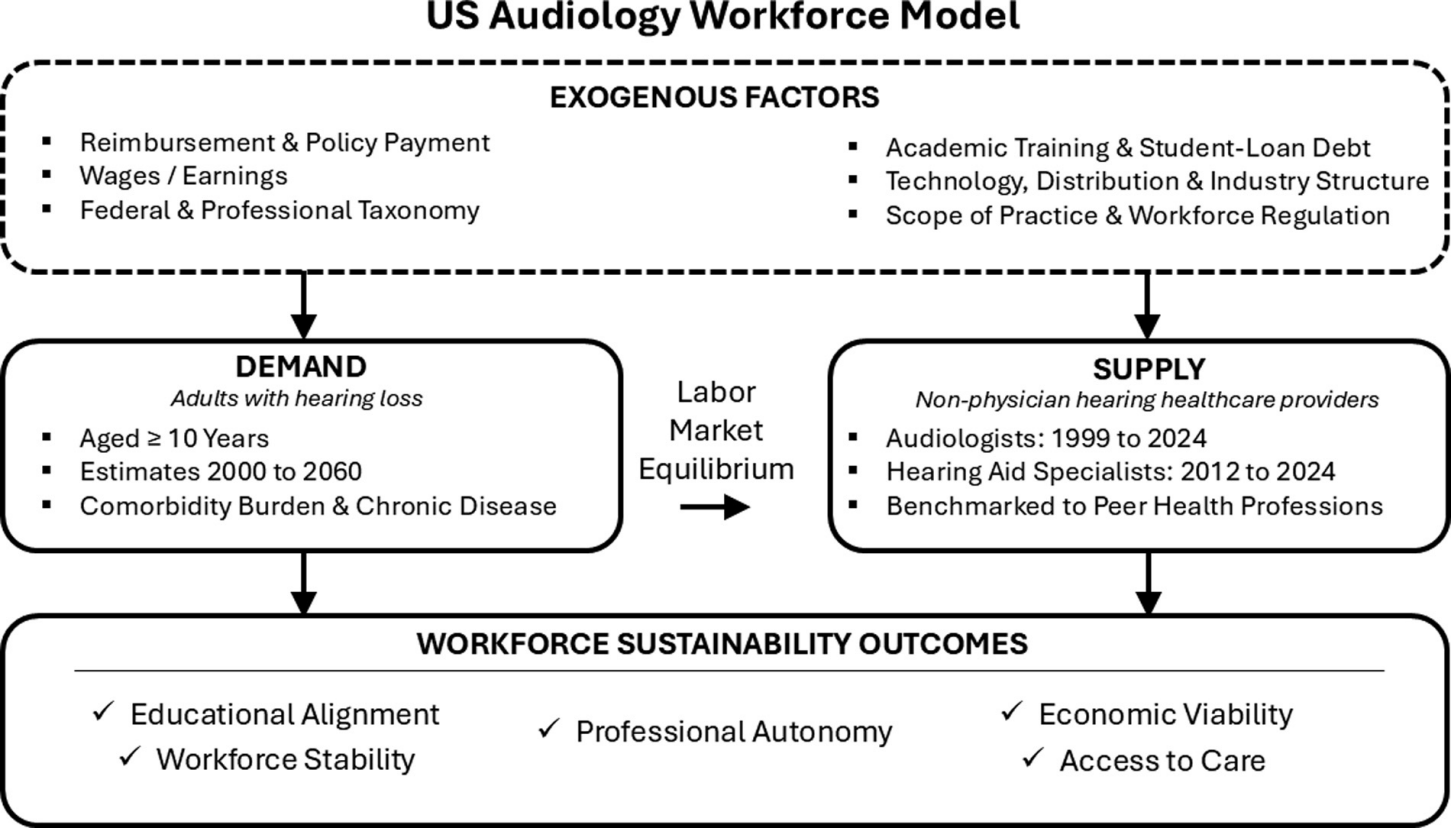
Model developed to evaluate the US audiology workforce and the structural determinants of professional autonomy.

The framework incorporates:

- Increasing demand for hearing and balance services among US population aged >=10 years of age between 2000 and 2060—capturing demographic aging, rising prevalence of chronic disease, population growth, and projected increase in sensory disorders.
- Provider supply, limited to the two non-physician occupations central to US hearing healthcare—audiologists and hearing aid specialists—allowing direct assessment of how the distribution, growth, and retention of these providers relate to population-level needs.
- Comparative analysis across other non-physician healthcare fields—important for evaluating audiology's relative workforce growth, labor market competitiveness, and alignment with broader healthcare trends.
- Exogenous forces that directly influence workforce viability, including:
  – Program graduation rates and educational pipeline variability,
  – Student loan debt and its impact on career choices, burnout, and early attrition,
  – Professional taxonomy and scope-of-practice restrictions across states,
  – Federal classification and reimbursement policies that limit or enhance autonomy, and
  – The ability of the profession to deliver services without unnecessary intermediaries.

In sum, this model provides a rigorous structure for evaluating how well audiology is positioned to meet population needs and whether current educational, economic, and regulatory structures are sufficient to sustain an autonomous workforce.

## Data Source and Analytical Approach

Some analyses in this issue draw primarily from the U.S. Bureau of Labor Statistics (BLS) Occupational Employment and Wage Statistics (OEWS) database, the nation's most comprehensive ongoing survey of employment and wage patterns across industries and occupations. The OEWS provides standardized, longitudinally consistent data, making it uniquely suited for studying workforce evolution over time.

A critical feature of the dataset is that audiology received its own Standard Occupational Classification (SOC) code in 1999, allowing for discrete identification of audiologists from speech-language pathologists (SLP). 9 This classification provides a clean baseline for evaluating long-term changes in employment, workforce distribution, and wage trends over a 25-year period.

Using OEWS data, this issue examines:

- Longitudinal workforce patterns from 1999 to 2024, capturing the full period since audiology's formal recognition as a standalone occupation.
- Focused comparative analyses from 2012 to 2024—the era following the widespread adoption of the AuD as the entry-level credential, when the profession's workforce, wage, and retention patterns would be expected to reflect doctoral-level preparation.
- Benchmark comparisons between audiology and other nonphysician healthcare professions (2012–2024) to contextualize audiology's labor trends within the broader healthcare labor market. The 2012–2024 window reflects BLS' 2012 revision of the SOC system, which expanded and reclassified numerous healthcare occupations – including the addition of hearing aid specialist as a distinct SOC code – enabling expanded cross-occupation comparison over this period
- Supplemental student pipeline analyses, including graduation data, wage trends, and demographic patterns drawn from national education and labor datasets to link workforce outcomes with educational system inputs.

Together, these analytical components offer a comprehensive view of how audiology's workforce has changed over time and how structural forces—economic, regulatory, and educational—shape current and future supply and constrain the profession's capacity for autonomous practice.

## Limitations

Although the BLS OEWS dataset represents the strongest available national source for longitudinal workforce analysis, several important limitations must be acknowledged when interpreting results:

- Point-in-time sampling: OEWS data reflect employment estimates from a single month each year, not continuous or real-time counts. Seasonal variations or short-term employment shifts may not be captured.
- Exclusion of self-employed clinicians: Private-practice owners and contract hire employees who make up a meaningful segment of the audiology workforce—are not included in OEWS estimates. This omission leads to a systematic undercount of total practicing audiologists.
- Macro-level reporting: Data are aggregated at the profession, state, and industry levels. They do not provide information on subspecialty practice patterns (e.g., vestibular, cochlear implants, tinnitus, pediatrics).
- Wage estimates reflect employer-reported data; compensation structures that rely heavily on commission, productivity incentives, or profit-sharing may be underrepresented.
- Occupational role ambiguity: In settings where audiologists also perform tasks performed by hearing aid specialists or other personnel, occupational misclassification can occur.

Because of these constraints, the goal of the workforce model is *not* to determine the exact number of practicing audiologists in the United States. Instead, the model is intended to:

- Identify directional trends over time,
- Benchmark audiology's growth relative to comparable health professions,
- Illuminate structural vulnerabilities in the workforce pipeline, and
- Inform policy, educational, and professional strategies that support future workforce sustainability.

Collectively, these limitations affect point estimates and absolute counts but do not undermine the model's ability to identify relative trends, comparative benchmarks, or structural patterns over time.

Finally, the analyses presented in this issue were completed during a period of active federal policy change. The One Big Beautiful Bill Act (OBBBA), enacted in 2025, introduces possible structural changes to graduate student lending—including aggregate borrowing caps and professional-degree classification criteria that currently exclude audiology—whose effects on workforce entry, program enrollment, and professional composition are not yet reflected in available data. Future workforce analyses will need to account for how these financing constraints interact with the earnings and classification conditions this issue documents.

## Articles in this Issue

The manuscripts comprising this special issue represent the culmination of a research program that began in 2019. Early iterations of these ideas were developed through conference presentations and professional convenings, and the work published here reflects the maturation of that ongoing scholarly dialogue.

This special issue includes eight contributions that collectively represent the most comprehensive workforce examination the profession has undertaken in decades. The eight articles that follow are not independent contributions ( Fig. 2 ). The issue opens with workforce supply (Audiology Workforce Supply and Population Demand) and earnings data (Economics of Audiology Wages) to ground the analysis in measurable outcomes before turning to the structural and policy explanations that that the following two articles provide (Audiology at the Intersection of Labor, Revenue, and Professional Taxonomy and Audiology's Nomenclature and Taxonomy Under the Law). The next two articles (Economic Implications of Audiology Student Debt for Workforce Sustainability and Shaping the Future of Audiology) then translate those structural conditions into their human consequences, before the summary two articles (Professional Status of Audiology in U.S. Healthcare and Audiology in 2050) synthesize the full body of evidence into a diagnosis and a reform roadmap. Readers will benefit from understanding this sequence before proceeding.

**Fig. 2:**
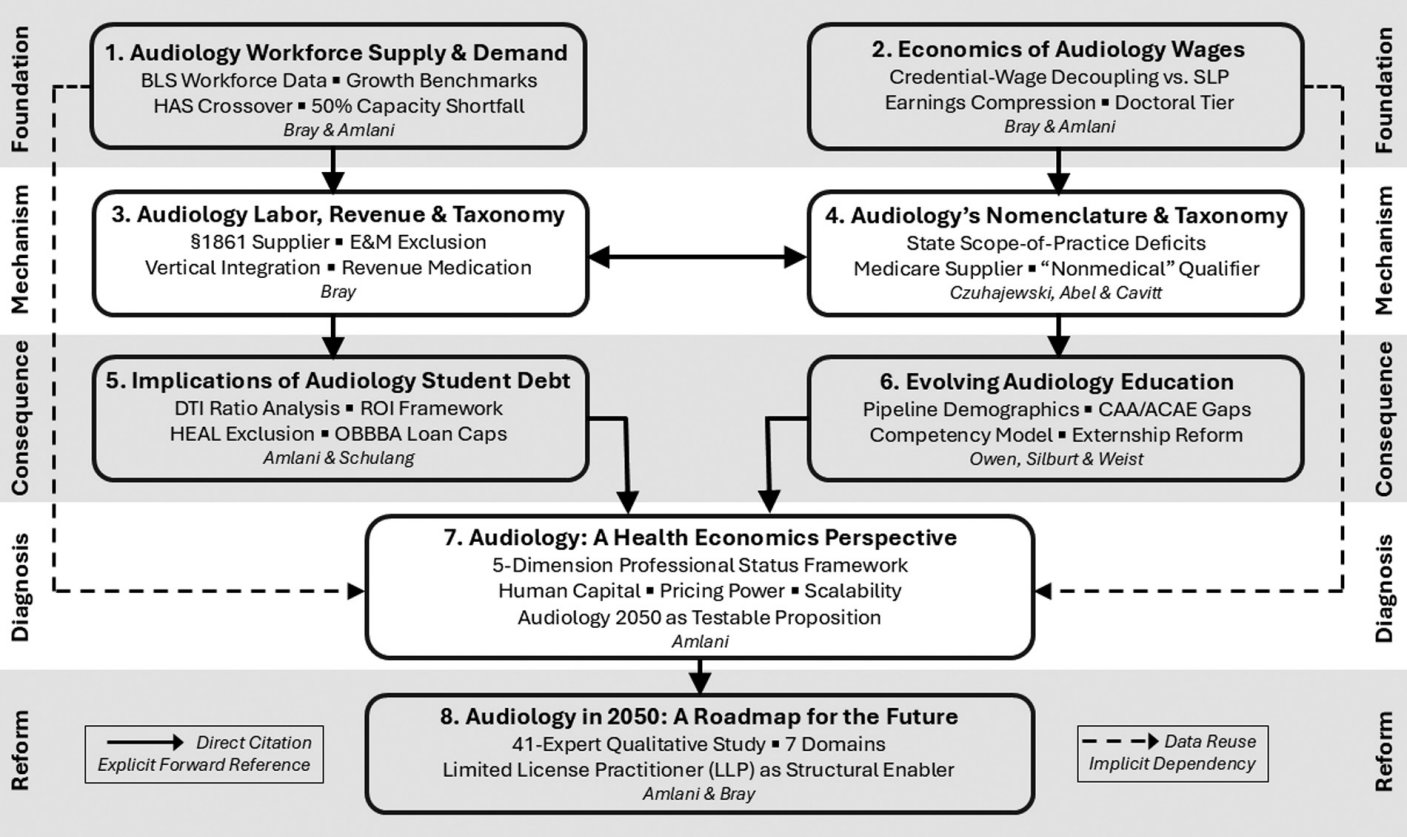
Cross-citation architecture of the special issue. Each article is organized within one of five structural layers—Foundation, Mechanism, Consequence, Diagnosis, and Reform—reflecting its role in the series' cumulative argument. Solid arrows indicate direct citation or explicit forward reference; dashed arrows indicate implicit dependency or data reuse. Articles 7 and 8 synthesize evidence from all prior contributions; the bold arrow from Article 7 to Article 8 represents the primary analytical spine of the issue. ACAE, Accreditation Commission for Audiology Education; BLS, U.S. Bureau of Labor Statistics; CAA, Council on Academic Accreditation; DTI ratio, debt-to-income ratio; E&M, evaluation and management; HAS, hearing aid specialist; HEAL, Health Education Assistance Loan; MAAIA, Medicare Audiology Access Improvement Act of 2025; OBBBA, One Big Beautiful Bill Act; ROI; SLP, speech-language pathologists.

**1. Audiology Workforce Supply and Population Demand: Trends, Benchmarks, and Implications (Bray and Amlani)**

The opening article establishes the empirical foundation for the special issue by examining the supply-side trajectory of the U.S. audiology workforce from 1999 to 2024—the full period during which audiology has been formally recognized by the Bureau of Labor Statistics as a distinct occupation. Using this 25-year window, the authors benchmark audiology's workforce growth against other non-physician healthcare professions, including optometry, occupational therapy, physical therapy, and speech-language pathology, as well as hearing aid specialist (limited to 2012–2024).

A central finding is that the transition to doctoral-level entry introduced a structural legacy effect on workforce capacity: a 27% workforce contraction between 2000 and 2004 was followed by a recovery that has not fully offset cumulative losses, leaving audiology with the third lowest growth rate among seventeen advanced–degree health professions. Analysis of graduate-to-workforce ratios further demonstrates that this constraint is not primarily one of educational output, but of downstream retention, utilization, and economic alignment. By modeling provider-to-population ratios from 2000 through projected estimates in 2060, the article documents a widening capacity gap that cannot be resolved through incremental workforce expansion alone—and that is already observable in the accelerating growth of the hearing aid specialist workforce, which is on trajectory to surpass audiology in total workforce size by around 2036.

Together, these findings frame the central question the issue addresses: why has a profession with a stable doctoral pipeline failed to achieve workforce growth commensurate with population demand and peer professions? The answer, this article suggests, lies not in educational supply but because audiology must have a greater departure rate than peer professions (where departure must include abandonment of the profession). To understand this, the structural conditions that govern how audiologic labor is valued must be investigated—conditions that the next article (Economics of Audiology Wages) examines through the lens of professional earnings.

**2. Economics of Audiology Wages: Benchmarking a Doctoral Health Profession Within U.S. Healthcare (Bray and Amlani)**

In healthcare labor markets, earnings function as a revealed signal of professional valuation—reflecting not stated intent or aspirational role definitions, but how employers, payers, and institutions actually price clinical expertise in practice. This article examines that signal for audiology by analyzing wage and earnings trajectories compared to speech-language pathology over the past 25 years and benchmarking current wages (2022-2024) against 34 healthcare professions including 10 Associate's degree, 6 Bachelor's degree, 9 Master's degree, and 9 Doctoral degree professions.

**3. Audiology at the Intersection of Labor, Revenue, and Professional Taxonomy (Bray)**

The first two articles document that audiology's workforce is constrained and its earnings are suppressed. This article examines why. Applying a labor economics and professional taxonomy framework, it identifies the institutional architecture—not insufficient training, patient need, or clinical scope—as the primary driver of the earnings and workforce outcomes the preceding analyses document.

The analysis proceeds through three integrated domains:

- At the level of professional taxonomy, audiology's designation under Section 1861 of the Social Security Act as a supplier of diagnostic services rather than a limited license practitioner produces three compounding constraints. First, it restricts independent billing authority and reinforces referral-dependent care models that decouple population-level demand for hearing care from institutional demand for audiologic services. Second, and most consequentially for professional valuation, it excludes audiologists from evaluation and management (E&M) codes—CPT 99202–99215—the billing mechanism through which diagnostician-centered professions capture the economic value of clinical assessment, interpretive reasoning, and care planning. Because audiologists routinely perform each of these functions, their exclusion from E&M billing means that a substantial portion of doctoral-level cognitive labor is performed without independent reimbursement recognition. Third, this classification places audiology outside the provider and practitioner categories through which federal policy expansions—including telehealth, value-based payment models, and alternative payment arrangements—are routinely extended to other professions.
- At the level of revenue generation, audiology occupies a hybrid position—simultaneously medical, retail, and therapeutic—without full economic recognition in any of those frameworks, and without the practice ownership patterns and pricing authority that allow structurally analogous professions such as optometry and veterinary medicine to convert product-linked care into scalable professional revenue. Compounding these constraints, vertical integration by major hearing aid manufacturers into third-party administrator networks, retail clinic chains, and direct-to-consumer platforms has created a market architecture in which independent audiologic practice competes for patients and reimbursement against entities that simultaneously control upstream supply, downstream distribution, and the benefit structures governing patient access.
- At the level of labor markets, these structural conditions produce the wage outcomes the second article (Economics of Audiology Wages) documents: earnings compression within the doctoral degree cohort, wage convergence with a master's-level profession despite a full degree of separation, and a workforce productivity ceiling bounded by one-to-one service delivery.

Underlying these conditions is a self-reinforcing structural cycle: because audiologists cannot independently bill for cognitive clinical labor at the doctoral level, product transactions become the primary revenue mechanism to sustain practice operations; because revenue remains anchored to product sales rather than independently priced clinical services, the profession is perceived—by payers, policymakers, and the public—as a retail rather than clinical enterprise; and because that perception persists, the billing reclassification that would break the cycle remains elusive. Correcting the taxonomy is not merely a regulatory objective, it is the mechanism through which this cycle can be interrupted.

The article concludes that durable improvement in audiology's economic position requires coordinated alignment across all three domains—taxonomy, revenue structure, and labor deployment—and that the regulatory infrastructure for this alignment is already partially in place. Audiologists hold individual National Provider Identification numbers, and Centers for Medicare and Medicaid Services (CMS) already requires services to be billed under the audiologist's own provider number. What remains is the reclassification that would convert partial recognition into full reimbursement authority. The next article documents the statutory and regulatory conditions—at both the state and federal levels—that this reclassification must address.

**4. Audiology's Nomenclature and Taxonomy Under the Law: Implications for Access and Coverage of Audiology Services (Czuhajewski, Abel, and Cavitt)**

While Audiology at the Intersection of Labor, Revenue, and Professional Taxonomy makes the economic structural argument for why audiology's classification constrains its workforce outcomes, this article provides the legal and statutory documentation. Drawing on a systematic review of state audiology practice acts, federal Medicare classification structures, and cross-profession regulatory comparisons, the authors audit the specific language through which audiology is defined—and, in important respects, constrained—within law and administrative policy.

The state-level findings are striking. Table 1 in the article presents a four-dimensional comparison of scope of practice authority –– the ability to order diagnostic testing, order imaging, prescribe medication, and supervise assistants –– across 10 clinical professions. Audiologists, despite holding a clinical doctorate, are authorized to perform none of these activities in the majority of states, standing alone among doctoral–level clinical professions in this respect. Most state practice acts further qualify audiology's activities with the term “nonmedical”—a designation that appears in no other comparable profession's statutory language and that carries direct consequences for payer recognition, interprofessional standing, and reimbursement eligibility. The origins of this language are traced to organized otolaryngology advocacy aimed at restricting independent audiologic competition, and its persistence represents an ongoing structural liability rather than a neutral historical artifact.

At the federal level, the article examines Medicare's classification of audiology services as diagnostic-only, which excludes rehabilitative, vestibular, and chronic-care services from coverage even when Medicare Part B authorizes them—creating the treatment vacuum that forces beneficiaries to pay privately for covered services or to access them through non-audiologist providers. Current legislation–the Medicare Audiology Access Improvement Act of 2025 (MAAIA)–examines the legislative mechanism through which this classification could be modernized, repositioning audiologists as limited license practitioners with direct access and expanded reimbursement authority across the full scope of contemporary audiology practice.

Together, the two articles examining the Intersection of Labor, Revenue, and Professional Taxonomy, and Audiology's Nomenclature and Taxonomy Under the Law establish that audiology's workforce and economic challenges are not incidental to clinical or educational choices but are structurally embedded in the legal and regulatory frameworks that govern how the profession is defined, classified, and compensated. The next two articles (Economic Implications of Audiology Student Debt for Workforce Sustainability and Shaping the Future of Audiology) then examine the human consequences of these structural conditions for the individuals who train for, enter, and practice within the profession.

**5. Economic Implications of Audiology Student Debt for Workforce Sustainability (Amlani and Schulang)**

The structural conditions documented in the prior two articles—constrained reimbursement authority, suppressed wages, and professional misclassification—do not operate in isolation. They interact with a third structural force: the cost of becoming an audiologist. This article examines educational debt not as an individual financial burden but as a systemic workforce input that shapes who enters the profession, where they practice, and how long they remain.

The analysis documents that AuD graduates carry among the highest graduate-level debt accumulation trajectories of any health profession benchmarked, with annual debt growth of approximately $3,515 per graduating year—second only to optometry. Applying a debt-to-income (DTI) ratio framework, the article demonstrates that at the median audiologist salary, an early-career AuD graduate retains approximately 19% of gross income as discretionary income compared with 25% for a comparably situated SLP graduate—and that this disparity worsens materially upon home purchase. Critically, this misalignment is not simply a wage-level problem correctable by incremental salary growth. It is a revenue-architecture problem: because audiologists lack the independently billable diagnostic identity that professions such as optometry possess, the ceiling on what graduates can earn is structurally constrained regardless of practice setting or clinical productivity.

Two federal classification failures compound this burden. First, when the Health Education Assistance Loan (HEAL) program was discontinued in 1998, its higher borrowing limits were preserved for programs historically eligible—but audiology, then a master's-level profession, was excluded. Despite the subsequent transition to doctoral-level entry, that exclusion has never been corrected, leaving AuD students with lower unsubsidized loan limits than peers in optometry, veterinary medicine, podiatry, and pharmacy. Second, the One Big Beautiful Bill Act (OBBBA) introduces aggregate federal borrowing caps that could apply to audiology while exempting programs classified as professional degrees under federal statute—a classification audiology does not currently hold. These converging financing constraints, the article argues, are not administrative oversights but expressions of the same professional misclassification that the prior two articles on Taxonomy and Nomenclature document across reimbursement and scope-of-practice policy.

The article closes by identifying the fourth-year externship as the highest-leverage near-term reform target: a year in which students pay full tuition while delivering revenue-generating clinical labor under externally licensed supervisors who receive no university compensation. Transitioning toward paid externship or residency-style models would reduce graduate debt by tens of thousands of dollars while simultaneously expanding training capacity and preceptor sustainability—the educational infrastructure that that the next article (Shaping the Future of Audiology Education) examines directly.

**6. Shaping the Future of Audiology: Current and Evolving Landscape of Audiology Education (Owen, Siburt, and Weist)**

While the articles on the Intersection of Labor, Revenue, and Professional Taxonomy, and Audiology's Nomenclature and Taxonomy Under the Law establish the external structural reforms audiology requires—modernized scope language, federal reclassification, expanded reimbursement authority—this article addresses the internal reform that must accompany them. A profession cannot credibly claim the clinical authority it seeks if its graduates are not consistently and fully prepared to exercise it. Educational design is therefore not peripheral to the profession's reform agenda, it is the foundation on which expanded scope, practitioner recognition, and durable workforce sustainability depend.

Drawing on longitudinal enrollment and degree-production data from the CSD Education Survey, accreditation standards from both the Council on Academic Accreditation (CAA) and the Accreditation Commission for Audiology Education (ACAE), and an analysis of pipeline demographics and educational structure, the article identifies persistent gaps between current training models and the competencies required for autonomous clinical practice. The applicant pipeline must be broadened and diversified, but outreach alone is insufficient without concurrent improvements to the profession's economic value proposition—a condition established in the articles Economics of Audiology Wages and Economic Implications of AuD Student Debt for Workforce Sustainability. Educational models must shift from opportunistic clinical exposure toward competency-based frameworks that produce consistent preparation across programs, including the diagnostic, managerial, and interprofessional competencies required for full-scope practice. The structure of clinical training—particularly the fourth-year externship—warrants reform to reduce the financial burden documented in the article on Economic Implications of AuD Student Debt, and accreditation standards must drive innovation rather than codify minimum compliance.

Critically, the authors frame interprofessional education not merely as good clinical preparation but as a structural investment in audiology's long–term professional standing: audiologists who are routinely integrated into multidisciplinary care teams occupy a position in which their services are recognized as medically necessary components of coordinated care—precisely the positioning that strengthens the case for Medicare practitioner reclassification and reinforces the scope-of-practice authority that that the articles on Audiology at the Intersection of Labor, Revenue, and Professional Taxonomy, and Audiology in 2050 identify as foundational to professional autonomy. Educational reform, in this framing, represents the profession's internal commitment to becoming what it is asking the healthcare system to recognize. The final two articles–Professional Status of Audiology in U.S. Healthcare and Audiology in 2050–then examine whether the healthcare system currently recognizes and values that professional evolution, and what coordinated structural alignment would be required for such recognition to occur.

**7. Professional Status of Audiology in US Healthcare: A Health Economics Perspective (Amlani)**

The preceding six articles document audiology's workforce and economic challenges from multiple angles. This article synthesizes that evidence into a unified diagnostic verdict, applying a structured health economics framework to assess what the combined findings reveal about audiology's present professional position—not as aspiration or self-description, but as reflected by observable market behavior.

The framework evaluates professional status across five interdependent dimensions: human capital investment and economic return; pricing and reimbursement authority; market power and substitutability; productivity, leverage, and scalability; and earnings and wage growth as a composite signal. Applied to audiology, the findings are consistent across all five dimensions. A 3-year mean annual salary of approximately $92,477, set against doctoral debt loads commonly exceeding $120,000, produces a debt-to-income ratio that compares unfavorably with the six-figure 3-year mean annual salary for optometry ($139,013) and physician assistants ($130,887)—professions with comparable or lesser training intensity. Reimbursement authority remains concentrated in a narrow band of diagnostic codes while rehabilitative and chronic-care services fall largely outside covered categories. Professional labor remains vulnerable to substitution through device-linked delivery models and vertically integrated benefit structures. And productivity is constrained to clinician-centric encounters without viable pathways for leverage or scale. Taken together, these signals place audiology in an intermediate professional position—neither a technical service occupation nor a fully autonomous doctoral profession in economic terms.

This diagnosis carries a specific implication for the reform agenda. This article identifies three economic thresholds audiology must cross before professional autonomy can be reflected in market behavior rather than merely asserted in statute or vision: federal practitioner recognition across the full scope of audiologic practice, not simply diagnostic carve-outs; a consistently reimbursed service extender pathway that decouples productivity from individual clinician time; and earnings trajectories that begin to widen meaningfully with experience, specialization, and practice ownership rather than remaining compressed across the clinical lifespan. Progress on any single threshold in isolation will produce limited effect. Audiology 2050, 10 interpreted through this economic lens, is not an aspirational vision, it is a specification of the structural alignment these thresholds require. The last article (Audiology in 2050) examines whether the profession's collective expert perspectives are organized around achieving that alignment.

**8. Audiology in 2050: Aligning Collective Professional Perspectives to Create a Roadmap for the Future (Analysis by Amlani and Bray)**

The concluding article asks a practical question: does the audiology profession—as represented by its clinical, academic, policy, and industry leaders—share a coherent understanding of what structural alignment requires, and is it organized to pursue it? To answer that question, the authors collected and analyzed open-ended survey responses from 41 subject matter experts, using the seven Audiology 2050 objectives as an analytic framework to evaluate alignment between collective professional perspectives and the structural reform agenda the preceding articles have assembled.

The expert findings converge on a clear sequencing logic. Scope modernization and standardized, evidence-based practice are not parallel objectives alongside federal reclassification and reimbursement reform, they are prerequisites for them. Respondents consistently identified the Veterans Administration model as proof of concept: audiologists functioning there with expanded clinical authority, independent entry points, and unrestricted care management demonstrate that the policy barriers to full-scope practice are the only remaining obstacle separating current audiology from a model that already demonstrably works at scale. The persistence of “nonmedical” statutory language, the absence of consistent assistant utilization models, and the variability of scope across practice settings were identified not as incidental limitations but as the specific conditions that prevent federal reclassification from functioning as intended even when achieved.

The article confronts directly the cost of inaction. Under status quo conditions—without coordinated scope modernization, without reimbursement reform, without educational alignment—audiology in 2050 is projected to occupy a progressively narrower role within hearing healthcare delivery: present, but with diminishing authority over how care is defined, delivered, and rewarded. Of note, new patient acquisition in private practice declined in 2025 for the first time on record despite continued demographic expansion, 11 and hearing aid specialist workforce growth is projected to reach parity with audiology around 2036. These are not projected risks, they are present-state conditions.

Importantly, expert respondents also identified a form of inertia internal to the profession itself: a segment of the audiology workforce that actively operates below its full doctoral scope by choice, and that may resist changes perceived as destabilizing to established practice models. Structural reform, however necessary, will remain insufficient if a meaningful portion of the profession opts out of the doctoral-level identity and responsibility that full autonomy requires. The article therefore concludes that realizing the Audiology 2050 objectives demand not only external policy change but also intentional internal culture change, and that the special issue—as a whole—provides the evidentiary foundation from which that coordinated effort must be launched.

## Clear Need

The early architects of the AuD movement laid the foundation for a free-standing, autonomous clinical profession and deliberately redirected audiology toward greater authority, accountability, and responsibility within healthcare. The analyses and perspectives presented in this special issue, however, make clear that the profession has not yet fully realized that vision. Persistent misalignment across education, workforce utilization, reimbursement, and federal classification continues to constrain the autonomy originally envisioned—and the evidence assembled here suggests that this misalignment is structural, not incidental, and will not resolve through incremental adjustment alone.

At the center of these interdependencies is a structural cycle in which the absence of doctoral-level billing authority forces product-dependent revenue models, which in turn reinforce the perception that audiology is a retail rather than clinical profession, which sustains the classification conditions that perpetuate the billing exclusion.

The series converges on a specific sequencing logic for reform. Scope modernization and standardized, evidence-based practice are not parallel objectives to be pursued alongside federal reclassification and reimbursement reform, they are prerequisites for them. Federal recognition that arrives ahead of scope clarity and educational alignment will be uneven in implementation and limited in impact. Educational reform that proceeds without corresponding changes to reimbursement architecture will prepare graduates for a professional role the healthcare system does not yet fully authorize. Reimbursement reform without workforce models capable of capturing its value will leave structural gains unrealized. The reforms this issue documents are interdependent, and they must be pursued in coordinated sequence rather than as isolated initiatives.

Modernizing educational models, expanding scope and service delivery pathways, aligning professional taxonomy with training and competency, integrating supervised service extenders, and advancing federal recognition consistent with doctoral-level preparation and autonomous clinical practice are not optional reforms. Together they represent the structural prerequisites for achieving the profession's intended autonomy and long-term workforce sustainability—and the seven articles that precede this closing article (Audiology in 2025) provides the evidentiary foundation from which that coordinated effort must be launched.

One further condition deserves acknowledgment. The analyses in this issue identify not only external structural barriers but also a form of inertia internal to the profession itself—a segment of the audiology workforce that actively operates below its full doctoral scope and that may resist changes perceived as destabilizing to established practice models. Structural reform, however necessary, will remain insufficient if a meaningful portion of the profession opts out of the doctoral-level identity and responsibility that full autonomy requires. The commitment this issue calls for is therefore not only political and legislative, but also cultural—and it must be embraced within the profession before it can be credibly claimed from the healthcare system.

Charting a sustainable course forward is not only a matter of professional identity or economic viability, it reflects an obligation to the patients and communities who depend on audiology for access to hearing and balance care across the lifespan. Honoring the legacy of those who advanced the AuD movement now requires more than vision alone. It requires shared commitment, coordinated execution, and the willingness to align the profession's structures—internal and external—with the level of responsibility it has already assumed.
